# TURN-IT: a novel turning intervention program to improve quality of turning in daily life in people with Parkinson’s disease

**DOI:** 10.1186/s12883-022-02934-5

**Published:** 2022-11-28

**Authors:** LA King, P Carlson-Kuhta, JL Wilhelm, JA Lapidus, ML Dale, LS Talman, N Barlow, M Mancini, FB Horak

**Affiliations:** 1 Department of Neurology, Oregon Health & Science University, Portland, OR USA; 2 School of Public Health, Oregon Health & Science University, Portland State University, Portland, OR USA; 3 APDM Wearable Technologies, a Clario Company, Portland, OR USA

**Keywords:** Parkinson disease, Turning, Physical rehabilitation, Falls, Inertial sensors

## Abstract

**Background:**

People with Parkinson’s disease (PD) have a high fall rate and many falls are associated with turns. Despite this, there is minimal research on effects of rehabilitation on the quality of turns. Further, quantifying turns in the home may have broader implications since rehabilitation of turns would ideally improve turning in real world mobility.

**Methods:**

Sixty people with PD and a history of falls will be randomized to receive either a novel TURNing InTervention (TURN-IT) or no intervention (control group). The TURN-IT group will be seen for 6 weeks (18 visits) for an individualized, progressive program that is based on the specific constraints of turning in PD. Wearable sensors will be used to measure 7 days of mobility, including turns, before and after intervention or control period. In addition, blinded assessments of gait, mobility and turns will occur before and after intervention for both groups and falls will be monitored for twelve months post intervention with bimonthly email questionnaires.

**Discussion:**

This study has the potential to change how we rehabilitate and assess turning in people with PD and falls. There are several novel aspects to our study including a comprehensive turning-focused intervention that is tailored to the underlying constraints that impair turning in people with PD. Further, our outcome measure of turning quality during 7 days of daily life is novel and has implications for determining real-life changes after rehabilitation. The ultimate goal of this rehabilitation intervention is to improve how patients turn in daily life and to reduce falls.

**Trials registration:**

This protocol is registered at clinicaltrials.gov; #NCT04897256; https://clinicaltrials.gov/ct2/show/NCT04897256?term=Horak&cond=Parkinson+Disease&draw=2&rank=4.

**Supplementary Information:**

The online version contains supplementary material available at 10.1186/s12883-022-02934-5.

## Background

Parkinson’s disease (PD) is responsible for more falls than any other chronic disease and imposes a heavy burden on over 3% of people over 65 years old [1]. Falls in PD often occur while turning and the majority of functional tasks in the home, such as cooking or navigating the bathroom, require multiple turns. In fact, over 40% of daily steps are turning steps [2]. In addition, when a fall occurs during a turn, it is 8 times more likely to result in a hip fracture since sideways falling to the ground may result in landing on the greater trochanter of the hip [3]. A video analysis of the most common circumstances of falls in daily life in 130 elderly people residing in long-term care revealed that that 41% of 223 recorded falls occurred during turning [4]. The majority of people with PD have difficulty turning, even early in the disease, because of the complex interaction of gait with balance during turning [5–8]. As a result, people with PD fall 5 times more than age-matched elderly [9].

Despite the large body of literature on walking, there is little research into the neural and biomechanical requirements to safely and effectively turn during daily life. Safe and effective turning essentially depends on the relationship between the body’s center of mass (CoM) and the base of support [10]. Transitioning from steady state linear patterns of gait to walk in a different direction requires complex biomechanical interactions that occur with specific timing [11–14]. In addition to the motor control requirements for turning, it is believed that different cognitive processes contribute to non-linear walking versus straight ahead walking. Specifically, cognitive function and set-shifting are thought to contribute to how older adults navigate a curved walking path, but not linear walking [15]. A systematic review on turning in older adults concluded that those with cognitive deficits had significantly longer walking times in both straight- and curved-path walking, and the relationship between performance and cognition was stronger for curved walking than for straight walking [16].

People with PD have unique constraints that affect their ability to turn. Even patients with mild PD who can quickly walk along a straight path without noticeable deficits in gait speed may show difficulties during turning [7.]. Effective turning requires specific skills such as axial flexibility, top-down coordination of eyes-head-trunk rotation, multisensory integration, motor planning, adequate motor preparation, attention and set-switching, all of which are affected by PD [12, 13]. Important deficits in people with PD that may impact turns include the inability to regulate step width during turning, rigidity and increased axial tone that interfere with neck and trunk rotation, bradykinesia leading to hypometric anticipatory postural adjustments (APA) and narrow stance width, and reduced attention (difficulty with dual task turning) [17–20]. Each of these constraints have the potential to be addressed using task-specific rehabilitation and may lead to improved turning during daily life and reduced falls. However, effective training protocols have not been established [21].

Rehabilitation of impaired turning in people with PD is an especially important consideration since studies have shown that many aspects of balance control are not improved with dopaminergic medication, despite improvements in straight-ahead gait [22]. Fewer studies have examined turning performance On and Off dopaminergic medications. One group that measured 180-degree turns On and Off dopaminergic medication using unplanned and preplanned turns (with a visual cue) found that people with PD in the On state increased their turning distance but did not increase axial rotation and still had difficulty regulating step width during turning, resulting in narrow, cross-over steps (the external foot crossed over the pathway of the internal leg) [23]. In addition, while levodopa medication improved the UPDRS and walking velocity, it did not improve turning stability, number of steps to turn, time to turn and en-bloc turning (timing of segmental rotations) [23]. Although our previous study [22] showed that levodopa significantly increased turning velocity in a group of 100 people with PD, it is unclear whether a faster turn is appropriate for people with PD or if faster turns result in more falls. In fact, we have shown that faster turns, without any training, resulted in a longer time spent with the body CoM outside the base of support compared to slower turns in people with PD [6].

Our laboratory has developed several constraint-focused interventions for PD including both the Agility Boot Camp (ABC) and the cognitive focused ABC (ABC-C) [24–27]. For this study, we have developed a turning rehabilitation program, TURNing InTervention (TURN-IT), that reflects our experience with both the ABC and ABC-C. These rehabilitation interventions are built upon a theoretical sensorimotor framework and rely upon impaired neural control systems in PD [25]. The present intervention, TURN-IT, follows a similar approach of incorporating multiple underlying constraints to effectively address turning and incorporates sensory and motor progressions.

Besides having very little guidance on turning rehabilitation for PD, we have previously lacked valid, accessible, objective measures of turning for clinical environments and during normal daily activities. Our laboratory, and others, have demonstrated that new wearable technology, involving small, wireless, inertial sensors, can be used to reliability quantify the duration, velocity and number of steps used to turn [28–30]. Recent work has demonstrated the feasibility of measuring quality and quantity of turning during daily life [28, 30, 31]. Furthermore, we demonstrated that quality (i.e. turn velocity), but not quantity (i.e. number of turns), of turning during daily life differentiates people with PD and age-matched control subjects [30]. Specifically, the percentage of the day subjects were walking or turning and the mean number of turns per day were not different in people with PD compared to healthy controls, but the quality of turning, such as turn velocity and number of steps to turn were significantly different between the PD and age-matched controls [30, 31]. Unfortunately, current clinical tests of balance and patient-reported outcomes do not reflect turning deficits in PD [5], highlighting the importance of new ways to quantify turning performance for future clinical trials.

Here, based on what we know about the underlying constraints on turning performance, we will test TURN-IT, a novel turning intervention for people with PD. We will examine which specific turning characteristics change with rehabilitation compared to no intervention. We hypothesize that turning intervention will improve turns in daily life and a no-intervention control group was chosen as a comparator to minimize confounding results from using an active comparator. We will use state-of-the-art daily-life monitoring with wearable sensors to measure change in turning performance in daily life. We will also explore the effect of TURN-IT on prospective falls.

## Methods and analysis

**Fig. 1:**
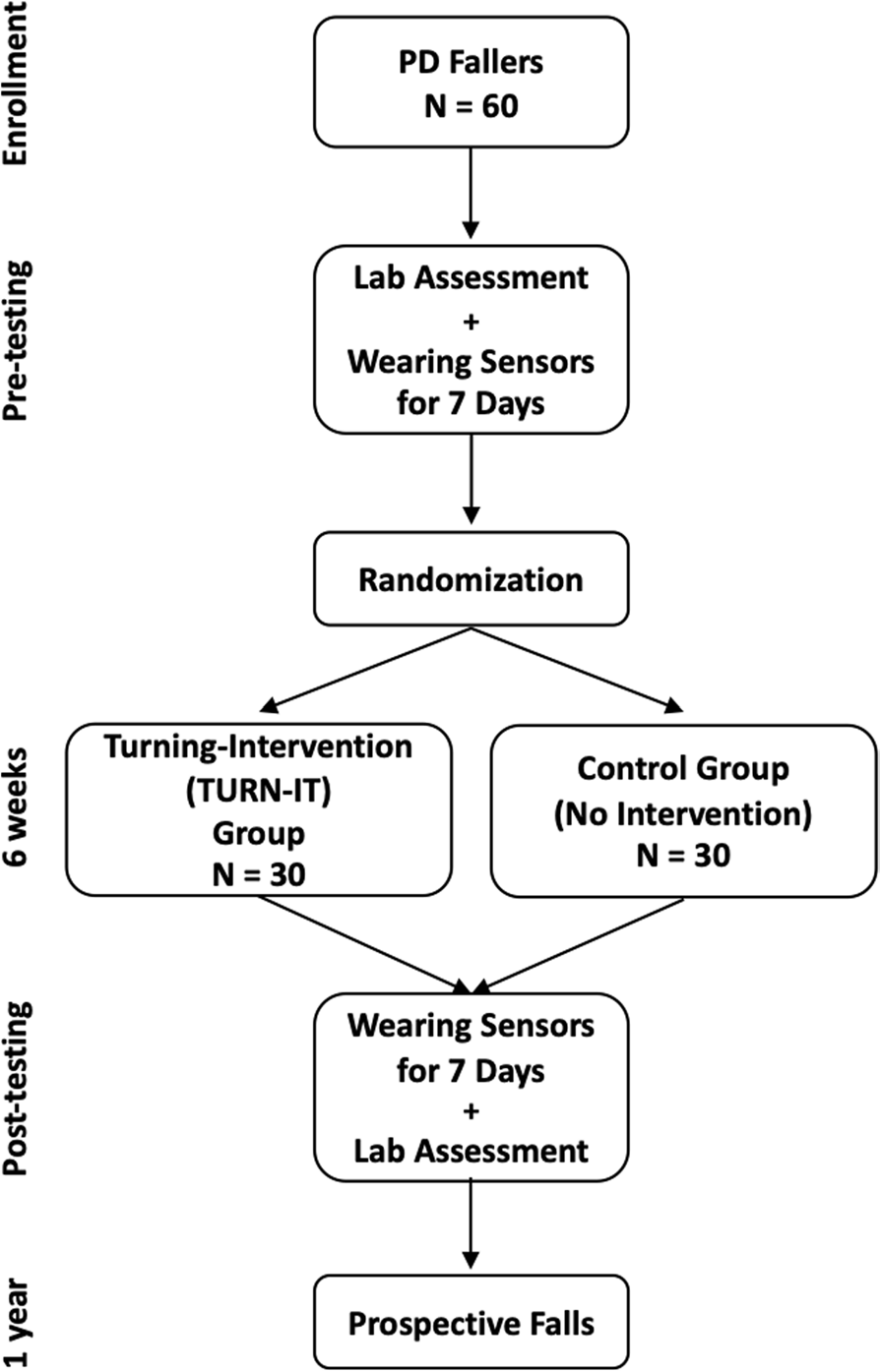
Flowchart of participant recruitment, randomization and testing

This study is part of a larger project investigating which set of objective measures of turning and gait during daily life best reflect fall risk. We will investigate which measures of turning and gait best discriminate fallers and non-fallers, as well as which measures can predict future falls. We will use daily-life monitoring of mobility as outcome measures for rehabilitation to improve turning. This is a randomized controlled trial that will compare people with PD who either undergo turning rehabilitation or are in a no intervention control group. Sixty people with PD and a history of falls will be randomized to a 6-week *TURN-IT* intervention (*n* = 30) or a Control *(*no intervention*)* group (*n* = 30). All participants will wear inertial sensors on both feet and on a belt for 7 days for daily-life monitoring immediately before and after the 6-week intervention. Falls will be tracked for 1 year prospectively using fall diary tracking with a strategy using bi-monthly automated emails and phone call follow-ups to ensure real-time tracking compliance (Fig. 1).

### Participants

*Inclusion criteria*: 1) Diagnosis of idiopathic PD from a movement disorders neurologist with the United Kingdom Brain Bank criteria of bradykinesia with at least one of the following—rest tremor, rigidity, and balance problems not from visual, vestibular, cerebellar or proprioceptive conditions; 2) responsive to levodopa; 3) Hoehn & Yahr [32] stages II-IV; 4) ages 55–85 years old, 5) self-report of at least one fall in the last 12 months, 6) willing and able to participate in exercise intervention sessions at Oregon Health & Science University (OHSU) campus, 3x/week for 6 weeks and refrain from changes in anti-Parkinson medications and activity levels during the intervention period. *Exclusion criteria:* 1) Major musculoskeletal or neurological disorders, structural brain disease, epilepsy, acute illness or health history, other than PD, significantly affecting gait and turning (*i.e.,* musculo-skeletal disorder, vestibular problem, head injury, stroke, cardiac disease, etc.); 2) no medical condition that precludes exercise; 3) Montreal Cognitive Assessment score (MoCA) ≤ 21 or inability to follow directions; 4) excessive use of alcohol or recreational drugs; 5) recent change in PD medication; and 6) inability to stand and walk for 2 min without an assistive device.

### Recruitment

Participants will be recruited by neurologists in the OHSU Movement Disorders Clinic and Portland Metro neurologists. We will also recruit during local outreach and education events and also on social media. For outside referrals, the diagnosis of idiopathic Parkinson’s disease will be confirmed by a movement disorder neurologist (MLD or LST).

### Sample Size

We will generate preliminary estimates of sensitivity to change in turning quality due to the TURN-IT intervention. These estimates ultimately will be used to inform power and sample size computations for a larger clinical trial. We examined effect sizes detectable by intervention at the planned sample size, and related these to effect sizes observed in other similar interventions. Our ABC study, which did not specifically focus on turning, resulted in a change in average turning peak speed of 1 standard deviation (SD) [33]. With the 60 subjects (30 randomized to each arm) we will have ample power (81.5% to 96.8%) to detect effects of 0.75 to 1 SD, and highly powered (> 99%) to detect 1.25 SD differences between groups at the 0.05 level of significance. We will also investigate the effects of the turning intervention on turning measures associated with fall risk (number of steps to turn, turn amplitude, trunk stability) and on the incidence of falls over a 12 month period after the intervention.

### Randomization for Rigor

After completing baseline testing, each participant will receive their group assignment from the unblinded research assistant. To maintain rigor of study design, subjects will be randomized into either the TURN-IT or Control groups via a blocked random allocation strategy. Randomization will use a blocked stratification design to balance the distribution of ‘PD severity’ (Hoehn and Yahr II or III-IV and freezing of gait status) between arms. The blocking will help ensure equal numbers in the two arms, and we will vary block sizes (2, 4, 6 or 8) to help avoid selection bias. All subjects will agree not to change their exercise activities or anti-Parkinson medication during the pre-intervention week of baseline testing, the 6-week intervention/control period or week of post-intervention week testing. The study biostatistician (JAL) will implement the randomization method in our Research Electronic Data Capture (REDCap) database [34].

### Blinding for Rigor

All pre- and post-testing will be carried out by researchers who are blinded to group assignment. Scientists analyzing daily-life monitoring data will be blinded to group assignment.

### Study Intervention

TURN-IT program*.* Participants in the treatment group will attend supervised, 1-h classes, 3 times per week for 6 weeks, one-on-one with the same exercise trainer, overseen by a physical therapist investigator. To allow participants to practice challenging, turning-focused exercises without risk of falling, we will use a ceiling-mounted motorized harness system. The minimal amount of body weight support (10%) will be used during the most challenging turning exercises, as this is required by the system when in use (ZeroG, Aretech, Ashburn, VA).

Table 1 summarizes the constraints from physiological systems due to PD that impair turning, the exercise principles aimed at each physiological constraint, example exercises and difficulty progressions. Training will be tailored to each participant’s impairments. Participants will rotate through four stations (Warm up walking, Axial rotation, Weight shifting and Turning; see Supplemental Table 1 Supplemental Fig. 1) that include multiple exercises designed to target the physiological constraints. The Supplemental Table 1 and figure gives exercise details for each station and progression of difficulty. The exercise trainer will record exercise progression and compliance for each subject on a weekly basis in the REDCap.

**Table 1:** Physiological constraints in Parkinson’s disease that affect turning. The table lists exercise principles, examples of exercises, and progressions of difficulty designed to improve turning ability

*Constraints*	*Exercise principles/Actions*	*Exercise examples*	*Exercise progressions*
***Narrow base of support***	Widen base of support with walking and turning at varying turn angles and speeds Avoiding crossover with sideways walking	Walking with turning at various degrees Figure 8 s around cones	Decreasing external cues (visual and verbal) for foot placement, increased speed
***Rigidity***	Decrease axial rigidity through axial mobility: improving trunk/pelvis flexibility Promote top-down approach for turning: eyes/head/trunk/pelvis coordination	Axial mobility exercises: trunk rotation, pelvic tilts Segmental turning	Progressing from mat table to floor, improve form and speed
***Bradykinesia***	Weight shifting with large APAs Use of metronome to increase speed of movement	Clock turns Rocking turns Lateral stepping over hurdles	Decreasing UE support, decreasing visual input with dribbling glasses, increasing speed, dual task
***Impaired sensory integration***	Kinesthetic awareness on body movement and foot placement Decrease visual dependence	Turning at various angles, navigation around obstacles and narrow spaces	Decreasing UE support, decreasing external cues (visual and verbal), adding hand and arm weights, decreasing visual input with dribbling glasses
***Reduced attention***	Dual tasks during mobility and turning exercises	Random callout on turns Adding cognitive dual-task activities Inhibition Go-No Go	Increased complexity of exercise, increased speed
***Inflexible set-switching***	Difficulty with functional mobility including sequencing	Rolling and bed mobility Sit to stand + turn	Decreasing visual cues, increased speed

*UE *Upper Extremity

#### Control group (no intervention)

Participants assigned to the control group will have no intervention and will be asked to maintain current activity levels (including no new exercise routines) and make no medication or activity changes during the 6-week intervention period as well as the pre and post intervention periods. To decrease chance of drop-outs in the control group, after the post-testing session, participants will be offered a meeting with the exercise trainer to review available community-based exercise programs that could be beneficial to them. Additionally, they will receive an extra $150 that could be used for these classes.

#### Assessment procedures

All people who are eligible per phone pre-screening will come into the OHSU Balance Disorders Laboratory for a screening visit. A member of the study team will verbally explain the consent form, allow the person ample time to read through the consent form and then will acknowledge informed consent by signing the form. After consenting and review of medical history, the Movement Disorders Society–Unified Parkinson’s Disease Rating Scale Motor Subscale III (MDS-UPDRS), the New Freezing of Gait Questionnaire (NFOG-Q), and the MoCA will be administered. If the participant is confirmed as eligible after the screening visit, they will be scheduled for a pre-testing assessment in the laboratory, after which randomization will occur. Immediately after the pre-testing laboratory visit, participants will be complete the seven days of daily-life monitoring and then start the 6-weeks of intervention or control. Following the 6-week intervention, participants will repeat the daily-life monitoring for 7 days and return for a post-testing laboratory session (see Fig. 1; Tables 2 and 3). All outcomes will be measured in the On levodopa state. All data will be stored securely in our REDCap database (clinical scales and questionnaires) and an Amazon Web Service (AWS) password protected secure server (home monitoring). Data integrity will be checked by a second person to ensure data quality. A formal data monitoring committee will not be used due to the small sample size and known minimal risk. Our team neurologist (MLD or LST) will be notified of all adverse events and will report to the Institutional Review Board(IRB) about any necessary change in protocol or safety concerns as needed. We do not foresee needing stopping rules since the risk of adverse events is low. Any required changes to the protocol or other significant aspects of the study will receive approval from our IRB and be updated in clinical trials as well as communicated to study teams and participants as appropriate.

**Table 2:** Summary of daily-life monitoring outcome measures

*Turning quality*	***Gait quality***	***Activity—mobility***
# steps to complete a turn	Stride velocity (cm/sec)	# turns per hour
^**a**^ Variability in # steps to turn Turn angle amplitude (deg)	Stride length (m) Cadence (steps/minute)	# steps per day # walking bouts per day
Turn peak velocity (deg/s) Turn duration (sec) Turn trunk jerk (m^2^/sec^2^)	Double support time (% gait cycle) & Variability Angle of foot at heel strike (deg) & Variability	Longest walking bout duration per day (sec)
	Lateral trunk range (degrees)	

^a^Primary outcome measure

**Table 3:** Clinical and fall-related outcome measures

*Name*	*Description*
Activities-Specific Balance Confidence Scale (ABC)	16-item self-report scale of confidence performing daily activities
Clinician Global Impression of Change (CGIC)	Clinician rating of mobility change
Falls Efficacy Scale-International (FES-I)	16-item questionnaire to assess fear of falling
Mini-Balance Evaluation Systems Test (mini-BESTest)	14-item assessment of 4 balance domains: anticipatory, reactive, sensory, and dynamic gait
Movement Disorders Society-Unified Parkinson’s Disease Rating Scale (MDS-UPDRS), part III	Clinical scale of severity of motor symptoms of Parkinson’s Disease
Parkinson’s Disease Questionnaire-39 (PDQ-39)	39-item self-report questionnaire to assess Parkinson’s disease specific health related quality in eight domains of quality of life
Patient Global Impression of Change (PGIC)	Patient rating of mobility change
Prospective Falls	Number of falls recorded for 12 months after end of intervention

#### Laboratory pre- and post-testing

In the laboratory, we will administer the short version of the Balance Evaluation Systems Test, MiniBESTest, a 14-item test of balance ability shown to be related to fall risk in people with PD [35, 36]. In addition, the following questionnaires will be administered: the Activities-specific Balance Confidence (ABC) Scale, the Falls Efficacy Scale International (FES-I) to assess fear of falling, the Parkinson’s Disease Quality of Life questionnaire (PDQ-39) to assess quality of life and the first 10 questions reflecting the Mobility domain for quality of life. Lastly, the Movement Disorders Society Universal Parkinson Disease Rating Scale motor subscale (MDS-UPDRS III) will be administered again at the post-intervention session as well as the Patient Global Impression of Change (PGIC), a one question measure on a seven point Likert scale in which the participant rates their perceived change [37] and the Clinical Global Impression of Change (CGIC) scale [37–39]. A blinded clinician (LST) will review videos of the MDS-UPDRS Part III and provide the score for the CGIC.

#### Daily-life monitoring

**Fig. 2:**
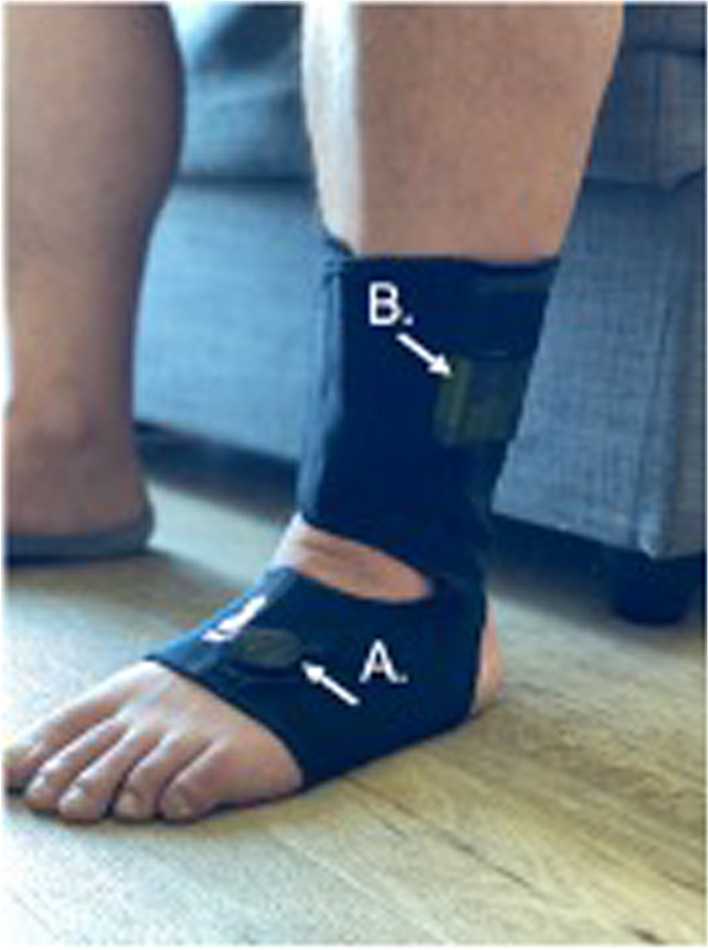
Instrumented socks with inertial sensors on top of foot (**A**) and battery on the side **B**

For measurement of mobility in daily life, participants will wear Opal Instrumented Socks that are made of a thin elastic cloth and have inertial sensors embedded. When worn, the sensors are located on the dorsum of the foot with the battery above the lateral malleolus (Fig. 2). They will also wear an Opal sensor on a belt with the sensor placed over the lumbar area (Opal and Opal instrumented socks by APDM Wearable Technologies, a Clario company). Participants will put the sensors on in the morning and wear them for 8–10 h per day for 7 days. The sensors are taken off and placed in a charger each night. At the end of the 7 days, the sensors are returned by mail or picked-up from their home by a study team member.

#### Primary outcome measure

To test our hypothesis that rehabilitation can improve turning performance, the primary outcome measure for this pilot study will be the change in variability (SD) of the number of steps to turn averaged over the 7 days of daily monitoring. Our previous studies showed that variability of the number of steps to turn was sensitive and specific to PD and related to falls in the elderly [30, 40].

*Secondary outcome measures* will be: 1) other daily-life gait and turning measures (Table 2), 2) daily life monitoring measures (Table 2), 3) clinical measures (Table 3), and 3) fall rate during 12 months after intervention.

#### Falls Tracking

After the post-testing session falls will be tracked prospectively for 12 months. Automated emails (via REDCap) will be sent every two weeks to ask if a fall(s) has occurred, and if there has been any change in medications or activity levels. If a participant responds YES to any of the questions, then a research assistant will follow-up with the participant for more details about the fall, change in medication or activity. If a fall has occurred, we will determine the severity and circumstances of the fall and whether it resulted in an injury or required medical intervention. A fall will be defined as “an event that results in coming to rest unintentionally on the ground or other lower level” [41].

### Statistical analysis

The primary outcome will be compared between the intervention and control groups after 6 weeks of participation in the trial. We will compare the two groups at the 6-week time point using analysis of covariance, controlling for individual baseline levels. Outcomes will be transformed to meet normality assumptions. Level of significance will be set at 0.05; however, the randomized group differences will be reported (regardless of significance), along with their corresponding 95% confidence intervals to inform power and sample size for future trials. Analyses will first be conducted without other covariates, then followed by models adding covariates (such as age, gender, disease duration, freezing of gait) to determine any attenuation or increase of group effect sizes. The change in outcomes from baseline to 6 weeks will be compared between groups via t-tests (no covariates), or linear regression (with covariates such as severity of disease, age, gender, freezing of gait) as well. The strategy for analysis of secondary outcomes will proceed similarly, although may require other types of regression models (logistic, ordinal, Poisson, etc.), depending on their measurement characteristics.

The effect sizes from models fit for this pilot study will be used to determine number of subjects required for a future clinical trial. Those effects sizes cannot be determined at this point, but as an example, we investigated sample size requirements under a variety of scenarios. As an example, if difference between groups after intervention is 0.50 SD after controlling for baseline levels, and baseline levels account for 20% of variability in outcome at follow-up, then we would require 52 patients per arm (104 total) in a two-group, parallel arm randomized trial to achieve 80% power when alpha = 0.05. As we intend to use this study to power future, larger study and investigate the efficacy of TURN-IT on mobility in daily life, we plan on continuing recruitment until we reach the abovementioned sample size and accommodate for any drop-out. No interim analysis on the primary outcome will be done as the size of the study is relatively small and due to the known minimal risk of exercise intervention for PD.

## Discussion

The TURN-IT study, a randomized controlled clinical trial, is designed to test whether a novel turning focused rehabilitation intervention can improve turns in daily life in people with PD who have a history of falls. There are several novel aspects to our study including a comprehensive turning-focused intervention that is tailored to each underlying constraint that impairs turning in people with PD. Further, our outcome measure of turning quality during seven days of daily life is cutting edge and has implications for determining real-life changes after rehabilitation. The ultimate goal of this rehabilitation intervention is to improve how patients turn in daily life and to reduce falls.

Previous work on turning rehabilitation is limited. The American Physical Therapy Association recently released the clinical practice guidelines (CPG) for physical therapy for PD [21]. This CPG is structured around 11 recommendations and turning is discussed within task-specific training, although only two studies were high quality and one was moderate quality. One high-quality study compared a turning training program using either a rotational treadmill or a turn-specific, training program and found that both the rotational treadmill program and turning-specific exercise group had greater turning improvement than the control program (seated stretching) [42]. The other high-quality study practiced turning in an aquatic setting and while they found improvements in the Timed Up and Go and Berg Balance scale, both of which include turning, quality of turning, itself, was not an outcome [43]. Another study of moderate quality used clock-stepping training as a cognitive movement strategy for turns and found that, compared to usual care, the turn training improved some measures of turning including speed and foot clearance but did not improve number of steps to turn [44].

There are a handful of other studies, not included in the CPG, that used different training approaches to improve turning. One study used augmented reality, visual cues for rehabilitation but found this approach did not help turning parameters and, in fact, worsened some [45]. Others have tried cueing with mixed results including a study that found unilateral auditory cueing and attentional strategies to prompt a turn with a head-first strategy did not improve axial movement deficits [46]. While the head-first strategy improved head-pelvic dissociation, it created other problems, including an exaggeration of the CoM shift to the inside of the turn. With cueing, turning became even more en-bloc with a decrease in separation of the head and pelvic rotations. Another small study focused on axial mobility to improve turns and showed improvements with a home-based exercise program [47]. The most recent Cochrane review of rehabilitation for PD summarizes studies showing improvements in gait speed but did not find rehabilitation studies to improve turning ability [48, 49]. We hypothesize that our intervention, which targets multiple underlying physiological constraints on turning performance, will improve daily life turns more than a singular intervention such as cueing.

An important aspect to this study is the focus on objective measures of turning in daily life as outcome measures. Our work and others has demonstrated the importance of home monitoring to detect deficits [50, 51] and this study will determine the role home monitoring of daily life as an outcome measure after rehabilitation. Leach et al. [52] also demonstrated the importance of continuous monitoring to measure turn quality, quantity and variability to prospectively determine fall risk in older adults.

The potential impact of effective turning rehabilitation for people with PD, and more generally in aging populations, may be profound. Falls are the most common reason for injuries in older individuals, and people with PD fall five times more than age-matched controls [9, 53]. Falls in the United States cost the economy an estimated 50 billion dollars in 2015 for both fatal and non-fatal falls in older adults [54]. In addition to the economic consequences, it has been show that falls can create fear of falling, which can restrict activity and negatively affect quality of life [55]. Turning is a task requiring control of dynamic balance that is impaired in many elderly, neurological, and orthopedic patients. Turning is often affected because it involves complex coordination of walking with dynamic balance control including an integration of multiple biomechanical, sensory and motor systems. Because turning is a dynamic balance skill that is critical for most functional activities, rehabilitation that can improve turning in daily life may improve independence, safety in daily activities and reduce falls.

## Supplementary Information

**Additional file 1: Supplemental Figure 1.** TURNing InTervention (TURN-IT) exercise stations with each focus and picture of representative exercises. The source of this image are photos from 2 participants in the study.

**Additional file 2: Supplemental Figure 2. **TURNing InTervention (TURN-IT) exercise progression.

## Data Availability

The data from this study will be made publicly available as supplementary materials in the publication.

## Abbreviations

ABC Scale: Activities-specific Balance Confidence Scale
ABC: Agility Boot Camp
ABC-C: Agility Boot Camp–Cognitive focus
APA: Anticipatory Postural Adjustments
APR: Automatic Postural Reaction
AWS: Amazon Web Service
CoM: Center of Mass
CGIC: Clinician Global Impression of Change
CPG: Clinical Practice Guidelines
FES-I: Falls Efficacy Scale International
IRB: Institutional Review Board
MoCA: Montreal Cognitive Assessment
MDS-UPDRS: Movement Disorders Society–Unified Parkinson’s Disease Rating Scale
NFOG-Q: New Freezing of Gait Questionnaire
OHSU: Oregon Health & Science University
PD: Parkinson’s Disease
PDQ-39: Parkinson’s Disease Questionnaire - 39
PGIC: Patient Global Impression of Change
REDCap: Research Electronic Data Capture
SD: Standard Deviation
TURN-IT: Turning Intervention
UE: Upper extremity
