# Supplementary figures and images for: TURN-IT: a novel turning intervention program to improve quality of turning in daily life in people with Parkinson’s disease
Source: BMC Neurol. 2022 Nov 28;22:442. doi: 10.1186/s12883-022-02934-5 (PMC9703770; doi:10.1186/s12883-022-02934-5)

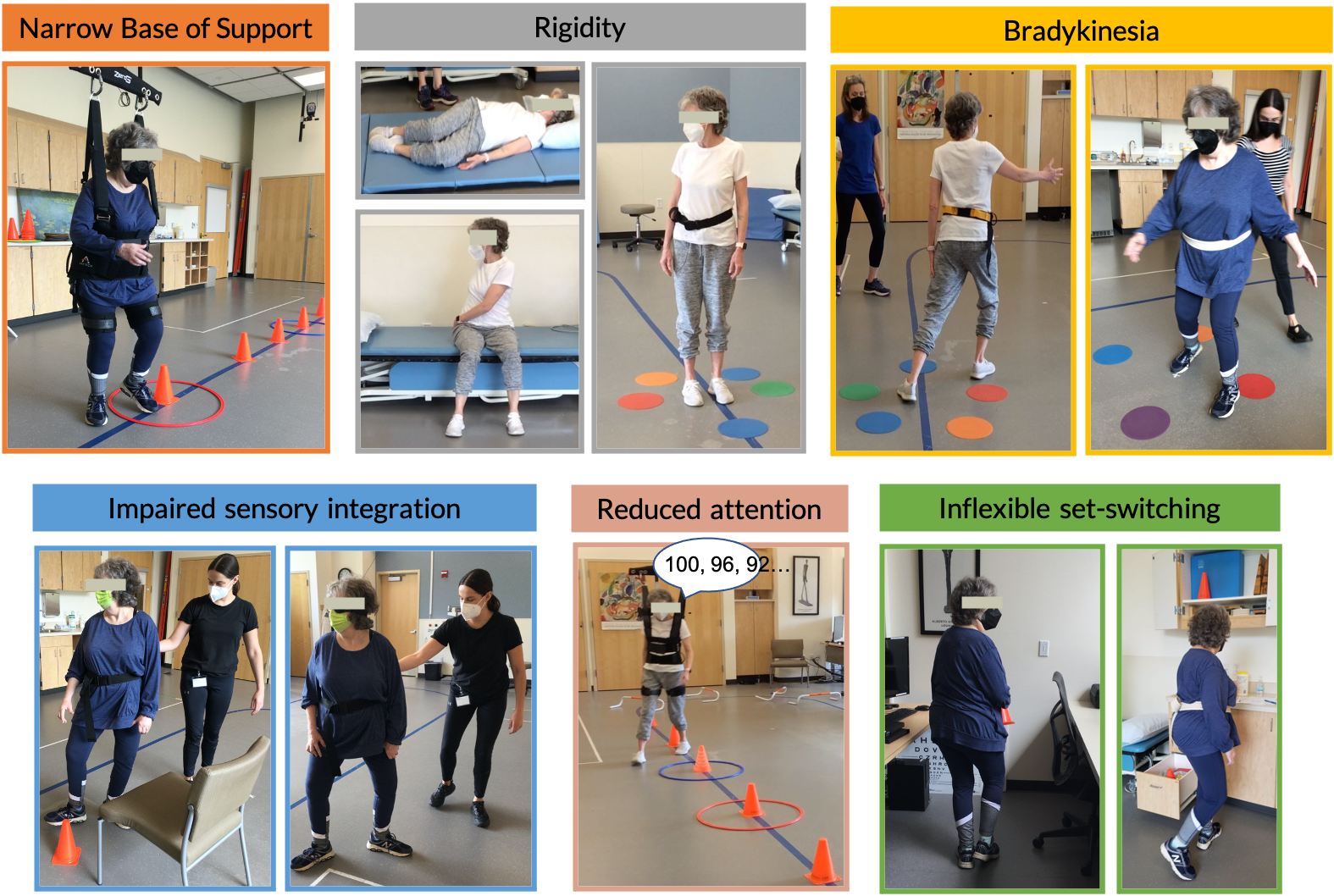

Supplement: Supplementary file 1 — Additional file 1: Supplemental Figure 1. TURNing InTervention (TURN-IT) exercise stations with each focus and picture of representative exercises. The source of this image are photos from 2 participants in the study. [file 12883_2022_2934_MOESM1_ESM.png]
