# Supplementary material for: TURN-IT: a novel turning intervention program to improve quality of turning in daily life in people with Parkinson’s disease
Source: BMC Neurol. 2022 Nov 28;22:442. doi: 10.1186/s12883-022-02934-5 (PMC9703770; doi:10.1186/s12883-022-02934-5)
Supplement: Supplementary file 2 — Additional file 2: Supplemental Figure 2. TURNing InTervention (TURN-IT) exercise progression. [file 12883_2022_2934_MOESM2_ESM.docx]

| Warm up (5-10 min): Walking over-ground progressing to treadmill | | | |
| --- | --- | --- | --- |
| Level | **Level of Support** | **Weights** | **Intensity**  **(Borg RPE 6-20)** |
| 1 | Harness, UE support | None | Light: 9-12/20 |
| 2 | Harness, no UE support | Hand weights | Hard: 13-16/20 |
| 3 | Gait belt | Hand and ankle weights | Very Hard: 17+/20 |
| Axial rotation* (15 min): Tasks include axial mobility exercise program progression including supine, seated and standing exercises, turning in place progressing from 45-360° | | | |
| Level | **Surface**  **(supine or seated)** | **Level of Support**  **(upright)** | **Sensory/Motor/**  **Cognitive Progression** |
| 1 | Mat table | Assistive device or UE support | Visual, verbal, auditory and tactile cues |
| 2 | Floor mat with use of chair for on/off floor | Gait belt | No cues or  dribbling glasses |
| 3 | Floor mat without use of chair for on/off floor | No Support | Dual task: cognitive |
| Weight shifting (10 min): Tasks include weight shifting in sitting and quadruped, clock stepping and functional exercises including reaching/twisting and sit to stand followed by a turn: | | | |
| Level | **Surface**  **(supine, seated or quadruped)** | **Level of Support**  **(upright)** | **Sensory/Motor/**  **Cognitive Progression** |
| 1 | Mat table | Assistive device or UE support | None |
| 2 | Floor mat with use of chair for on/off floor | Gait belt | Dribbling glasses |
| 3 | Floor mat without use of chair for on/off floor | No Support | Dual task: cognitive |
| Turning (20 min): This includes turns of varying angles, speeds and base of support. The tasks include walking with head turns, navigating obstacles, multi-directional walking, pivot turns, and an agility course. | | | |
| Level | **Level of support** | **Speed**  **(Metronome)** | **Sensory/Motor/**  **Cognitive Progression** |
| 1 | Harness, UE support | No pacing | Visual and verbal cues |
| 2 | Gait belt | Increase baseline speed by 20% | No cues or dribbling glasses |
| 3 | No Support | Increase baseline speed by 40% | Dual task: cognitive |

*Adapted from: Julie Chandler, M. S., PT Kathy Cotter Laub, Helga MacAller, Margaret Schenkman, and PT Julie Keysor. "AXIAL MOBILITY EXERCISE PROGRAM." And Schenkman, Margaret, Toni M. Cutson, Maggie Kuchibhatla, Julie Chandler, Carl F. Pieper, Laurie Ray, and Kathryn C. Laub. "Exercise to improve spinal flexibility and function for people with Parkinson's disease: a randomized, controlled trial." Journal of the American Geriatrics Society 46, no. 10 (1998): 1207-1216.
